# From Survival to Lifelong Care: The Global Burden of Congenital Heart Disease

**DOI:** 10.5334/gh.1580

**Published:** 2026-08-20

**Authors:** Raman Krishna Kumar, Mariachiara Di Cesare, Shreya Shrikhande, Elle Pendrick, Marvellous Adeoye, Natalie Evans, Bistra Zheleva, Tanya Hall, Babar Hasan, Nana-Akyaa Yao, Sarah Hamidi, Melissa Amato, Rachel Schofield, Lisa Hadeed, Pablo Perel, Sean Taylor, Jagat Narula, Daniel Piñeiro

**Affiliations:** 1 Amrita Institute of Medical Sciences and Research Centre, Kochi, Amrita Vishwa Vidyapeetham School of Medicine, India; 2 Institute of Public Health and Wellbeing, University of Essex, Colchester, United Kingdom; 3 World Heart Federation, Geneva, Switzerland; 4 Hearts4heart, Australia; 5 Children’s HeartLink, United States; 6 Division of Cardio-thoracic Sciences, Sindh Institute of Urology and Transplantation (SIUT), Pakistan; 7 National Cardiothoracic Centre & Korle-Bu Teaching Hospital Accra, Ghana; 8 St Bartholomew’s Hospital, Barts Health NHS Trust, London, United Kingdom; 9 Department of Non-Communicable Disease Epidemiology, London School of Hygiene & Tropical Medicine, London, United Kingdom; 10 University of Texas Health Science Center at Houston, UTHealth Houston University of Texas, Houston, TX, United States; 11 Department of Medicine, University of Buenos Aires, Buenos Aires, Argentina

**Keywords:** congenital heart disease, global burden of CHD, Health Equity

## Abstract

Congenital heart disease (CHD) is the most common congenital anomaly and remains an under-recognised contributor to the global burden of cardiovascular disease. Once regarded as a paediatric condition, its burden now extends across the life course, as growing numbers of patients survive into adulthood and require lifelong, specialised care. Here we examine the global burden of CHD and propose actionable strategies. Data from the Global Burden of Disease (GBD) study, supplemented by peer-reviewed literature, global reports, and expert input, were used. We examined trends in CHD incidence, prevalence, mortality, and disability-adjusted life years (DALYs), from 1990 to 2023. In 2023, an estimated 2.3 million children were born with CHD, and 16 million people were living with the condition worldwide, up from 11.8 million in 1990, with the largest increase in lower-middle-income regions. CHD has remained the leading cause of neonatal and infant mortality among non-communicable diseases across all regions since 1990. Age-standardised mortality reached 4.7 deaths per 100,000 people globally in 2023, remaining four times higher in low-income (5.7) than in high-income (1.4) regions. Profound inequities persist across the accessibility, capacity, quality, and affordability of care, as high-income countries have 140–180 times as many cardiac surgeons as low-income countries. Coordinated initiatives such as Kerala’s Hridyam programme have shown that meaningful gains are achievable even in resource-limited settings. Persistent care disparities are driven less by an absence of clinical knowledge than by unequal access to timely diagnosis, treatment, and lifelong follow-up. Addressing the global CHD crisis will require coordinated efforts from policymakers, healthcare systems, and global health organisations, supported by the proposed 2027 World Health Assembly resolution on childhood-onset heart disease.

## Graphical Abstract

**Figure:**
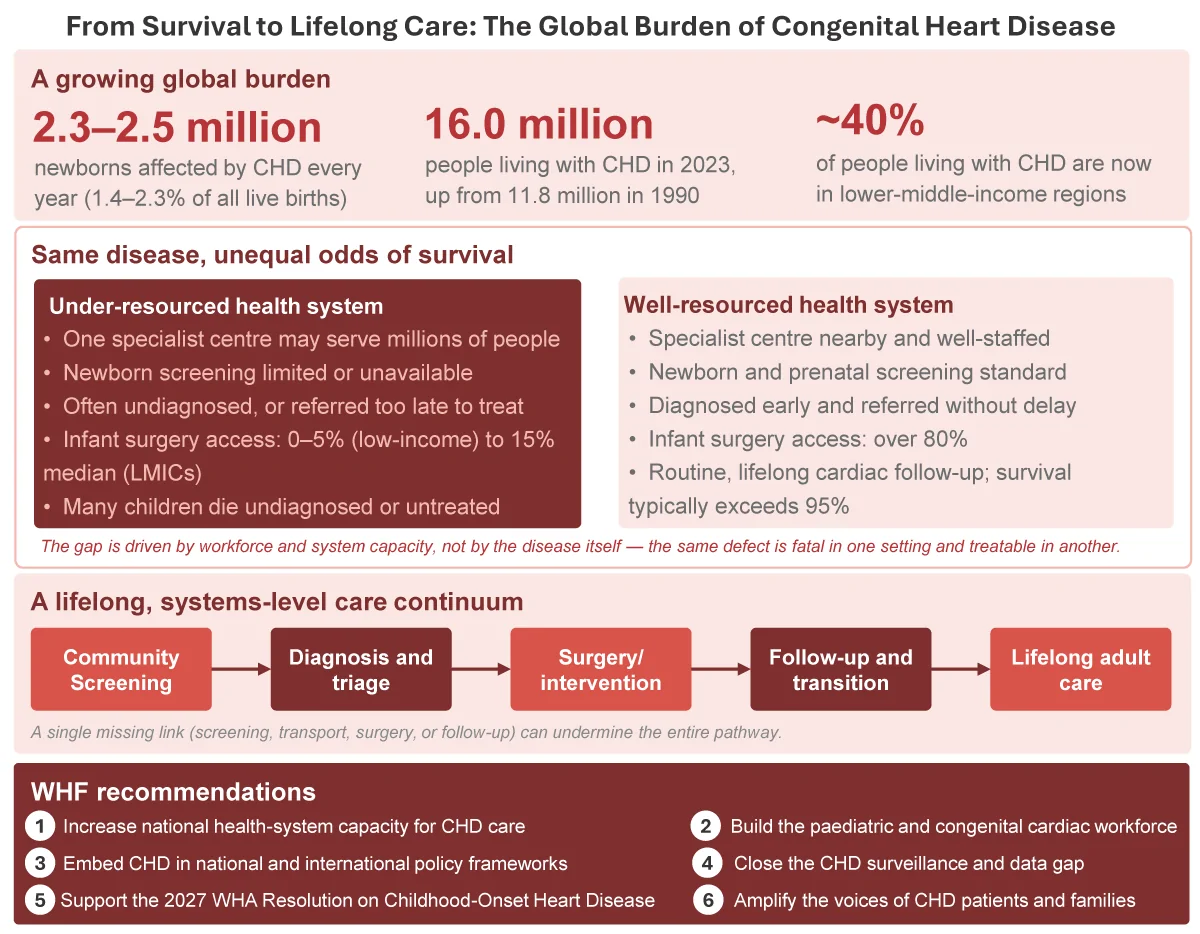


## Introduction

Congenital heart disease (CHD), a spectrum of structural abnormalities of the heart and great vessels present at birth, is the most common congenital anomaly and remains an unrecognised major global public health challenge (1,2). In 2023, an estimated 2.3 million new CHD cases occurred worldwide, bringing the total number of people living with CHD to nearly 16 million (3). Although CHD has traditionally been viewed as a paediatric condition, its burden now extends across the life course and increasingly reflects the broader global transition in disease patterns (4).

The burden of CHD extends across the lifespan. Infants and newborns in particular face high burden, with CHD ranking among the leading causes of infant mortality in most regions worldwide. This is especially evident in large populations within emerging economies/countries, where the most severe forms of CHD are often fatal without timely intervention that requires specialised infant and newborn heart surgery (5). However, advances in diagnosis and treatment have led to a profound demographic shift leading to an increasing number of children with CHD now surviving into adulthood following surgical correction or palliation, both in high-income and low- and middle-income countries. These patients require lifelong, nuanced care and pose unique challenges to all health systems (6,7).

Based on this shift, the growing population of adults with congenital heart disease presents additional complexity. There have been improvements in surgical care that have enabled survival, yet, these individuals face residual lesions, arrhythmias, heart failure, pulmonary hypertension, and in low- and middle-income countries (LMICs), often present in adulthood with uncorrected or late-repaired disease (4). For women with CHD, the reproductive journey presents challenges, including heightened risks during pregnancy, complex decisions about family planning, and managing cardiovascular transitions through menopause (8,9).

Despite its growing importance, CHD has not featured prominently in the global cardiovascular agenda. Its relative contribution to morbidity and mortality is likely to increase further as deaths from other readily treatable childhood conditions continue to decline in many settings. CHD therefore represents not only a paediatric survival issue, but also a lifelong condition with important implications for disability, quality of life, social participation, and health system planning.

The World Heart Federation (WHF), through its global network, is committed to addressing this crisis (10). Here we summarise the evidence and analysis underlying the World Heart Report 2026 (11), integrating epidemiological trends with health-system, economic, and lived-experience evidence to situate the global burden within the continuum of care. The report examines global trends in CHD epidemiology, explores geographic and socioeconomic disparities, identifies key determinants of outcomes, evaluates clinical and health system interventions, and proposes actionable strategies. By doing this, we aim to support cross-sectoral policy action and inform the development of evidence-based strategies for CHD prevention, early detection, treatment, and lifelong management.

## Methods

This paper draws on both secondary data and evidence synthesis. We relied on epidemiological data from internationally recognised sources to characterise the global burden of CHD, selected based on methodological robustness, completeness, and availability of disaggregated data by age, geography, and socioeconomic context. Estimates of CHD incidence, prevalence, mortality, and disability-adjusted life years (DALYs) were obtained from the Global Burden of Disease (GBD) (3). These data were analysed at global, regional, and country levels, with stratification by World Bank income classification (low, lower-middle, upper-middle, and high-income regions). Additional evidence on CHD subtypes, determinants, clinical outcomes, and health system factors was informed by published literature, global reports, and existing epidemiological studies. This was used to contextualise quantitative findings and support interpretation of patterns observed across regions. The analytical approach was primarily descriptive. We examined age-standardised incidence, prevalence, mortality, and DALY rates across regions and income groups. Temporal trends were assessed from 1990 to 2023, and geographic variation was evaluated at both regional and country levels. Comparative analyses were conducted to assess disparities in CHD burden across socioeconomic contexts. Age-standardised rates were used throughout to enable comparison across populations with differing age structures.

Beyond the epidemiological analysis, we synthesised evidence on the continuum of care, health-system requirements, economic burden, and policy from peer-reviewed literature, global and national reports, and structured input from clinical and public-health experts contributing to the World Heart Report 2026.

## Results

### The global burden of CHD: prevalence, mortality, and disability

**Figure 1:**
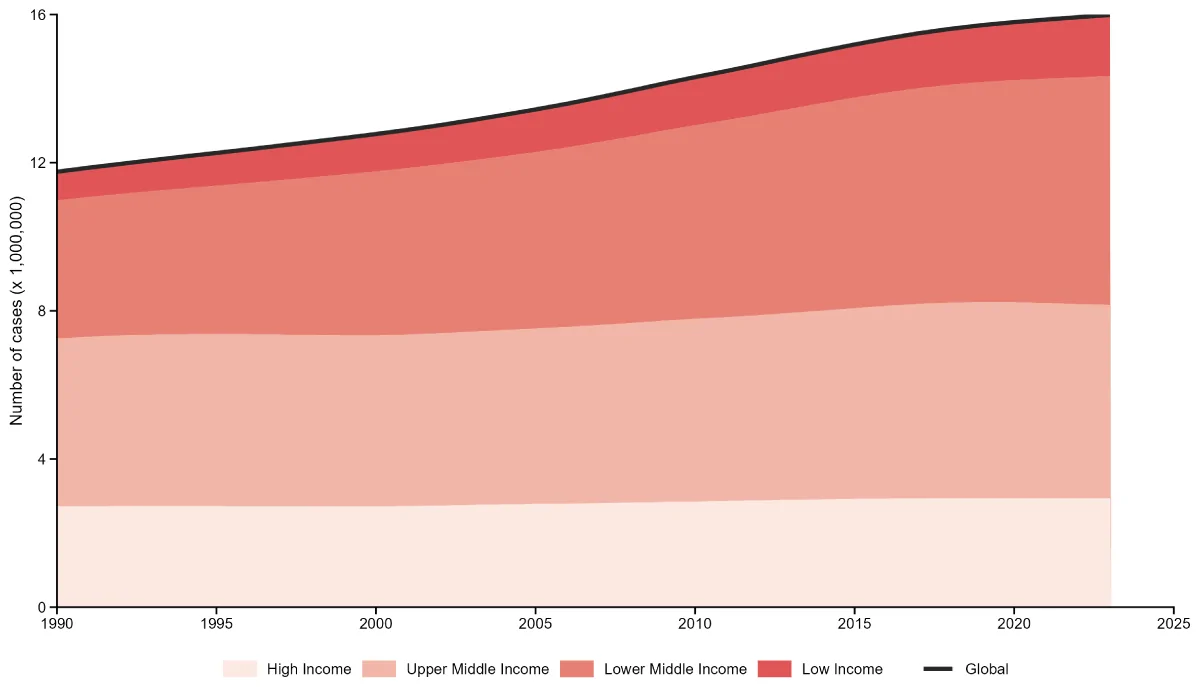
Number of people living with CHD by income regions, 1990–2023.

**Figure 2:**
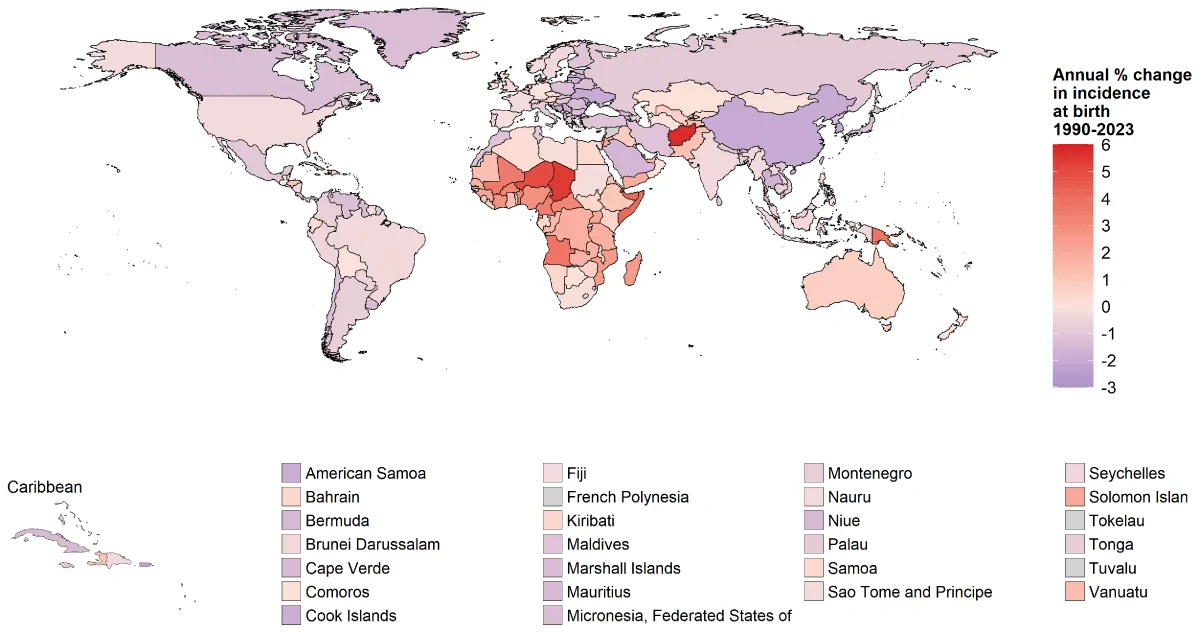
Annual percentage change in the CHD incidence rate, 1990–2023.

**Figure 3:**
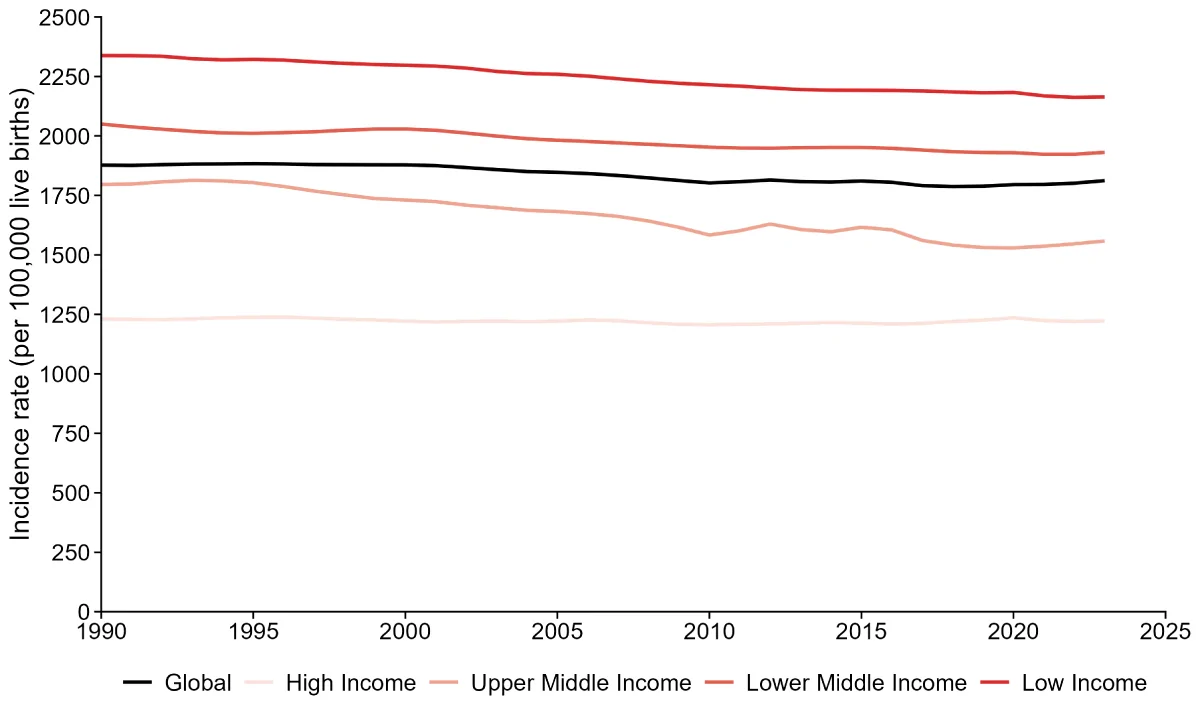
CHD incidence rate by income regions, 1990–2023.

**Figure 4:**
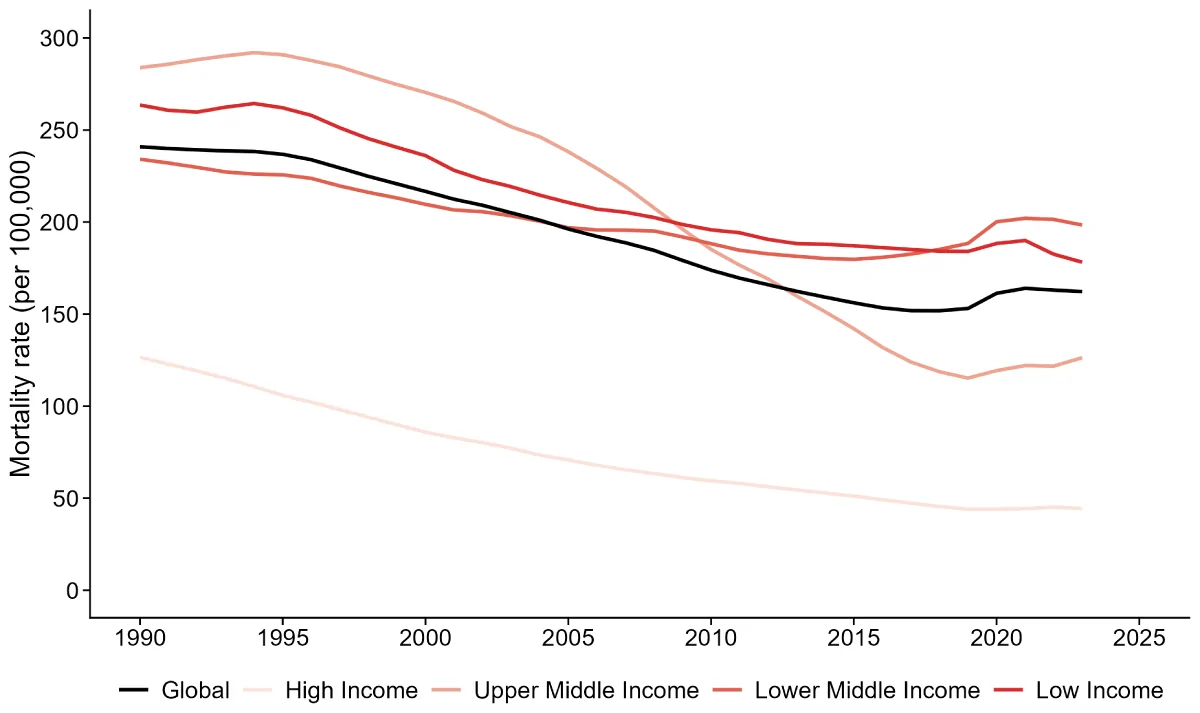
CHD infant mortality rate per 100,000 live births by income region, 1990–2023.

**Figure 5:**
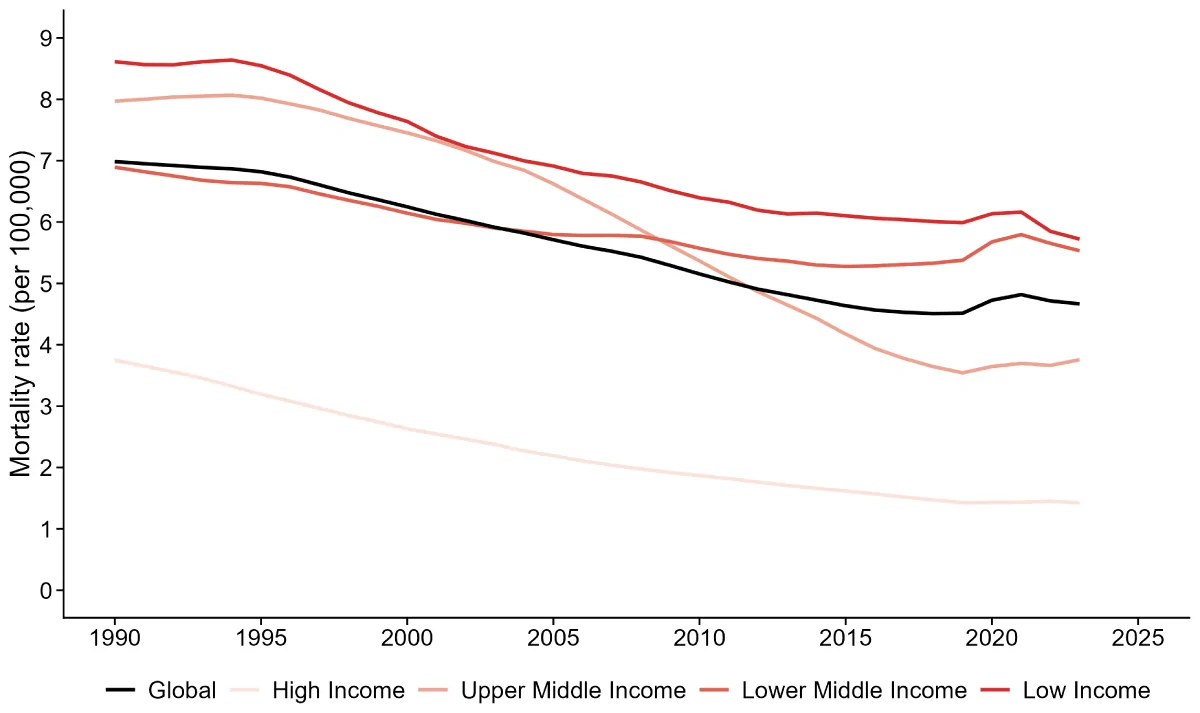
Age-standardised CHD mortality rate by income region, 1990–2023.

**Figure 6:**
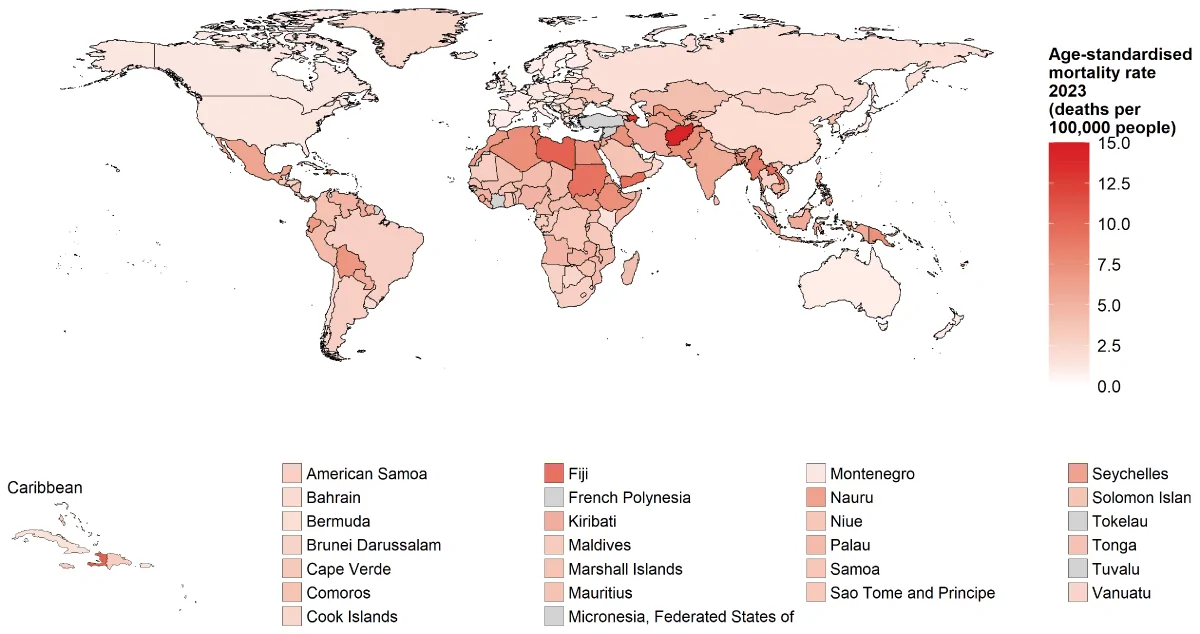
Age-standardised mortality rates, 2023.

Globally, an estimated 2.3–2.5 million newborns are affected by CHD each year, which represents approximately 1.4–2.3% of all live births. This is higher than conventionally reported estimates (2), perhaps reflecting the greater use of echocardiography and inclusion of a higher proportion of conditions such as atrial septal defect and bicommissural aortic valve. Progress in early diagnosis and more efficient surgical treatments has led to improvements in survival rates raising the number of people living with CHD from 11.8 million in 1990 to 16.0 million in 2023, with the largest increase in lower-middle-income regions, where nearly 40% now reside (Figure 1). The incidence rate has remained unchanged across regions (Figure 2), highest in low-income and lowest in high-income regions. Between 1990 and 2023, the average annual percentage change in CHD incidence ranged from –2.2% to +5.7%. The largest increases (above 4%) were recorded in Somalia, Djibouti, Niger, Chad, and Afghanistan, with sub-Saharan Africa being the region characterised by an increase in CHD incidence (Figure 3). CHD has remained the leading cause of neonatal and infant mortality among all non-communicable diseases across all regions since 1990, ranking second among all causes of infant mortality in high- and upper-middle-income regions (Table 1). Levels of infant mortality associated with CHD have consistently declined since 1990, reaching a mortality rate of 162.2 per 100,000 live births globally (Figure 4). Countries in upper-middle-income regions experienced the largest decline, from 283.9 per 100,000 live births in 1990 to 126.3 per 100,000 live births in 2023. All regions except high-income regions experienced a halt in the decline in 2020 as a result of the COVID-19 pandemic, with levels not yet returned to previous historical trend lines (Figure 4). Age-standardised mortality reached 4.7 deaths per 100,000 people globally in 2023, remaining highest in low-income regions (5.7 deaths per 100,000) and four times lower in high-income regions (1.4 deaths per 100,000) (Figure 5). The fastest decline occurred in upper-middle-income regions, where mortality more than halved; yet no region had reached high-income 1990 levels by 2023. Country-level mortality was highest in Afghanistan (14.1 deaths per 100,000), Azerbaijan (12.7 deaths per 100,000), and Libya and Haiti (both 10.5 deaths per 100,000) (Figure 6). In 2023, CHD accounted for 1.0% of total DALYs globally, down from 1.4% in 1990. Globally, the age-standardised DALY rate for CHD declined from 613.3 per 100,000 people in 1990 to 413.7 per 100,000 people in 2023. The low- and lower-middle-income regions recorded similar levels in 2023 (495.8 per 100,000 people and 487.8 per 100,000 people, respectively). These values were nearly four-fold those recorded in high-income regions (133.3 per 100,000 people).

**Table 1:** Ranking of congenital heart disease among leading causes of death, 1990 and 2023.


REGION	INFANT MORTALITY 1990 (<1 YEAR)	NEONATAL MORTALITY 1990 (<28 DAYS)	INFANT MORTALITY 2023 (<1 YEAR)	NEONATAL MORTALITY 2023 (<28 DAYS)

Global	7	8	6	6

High-income	2	3	2	4

Upper-middle	5	6	2	4

Lower-middle	10	9	5	6

Low-income	10	11	8	7


Values are ordinal rankings among all causes of death, not rates; a lower number denotes a higher rank. Data source: Institute for Health Metrics and Evaluation, Global Burden of Disease Study 2023.

**Figure 7:**
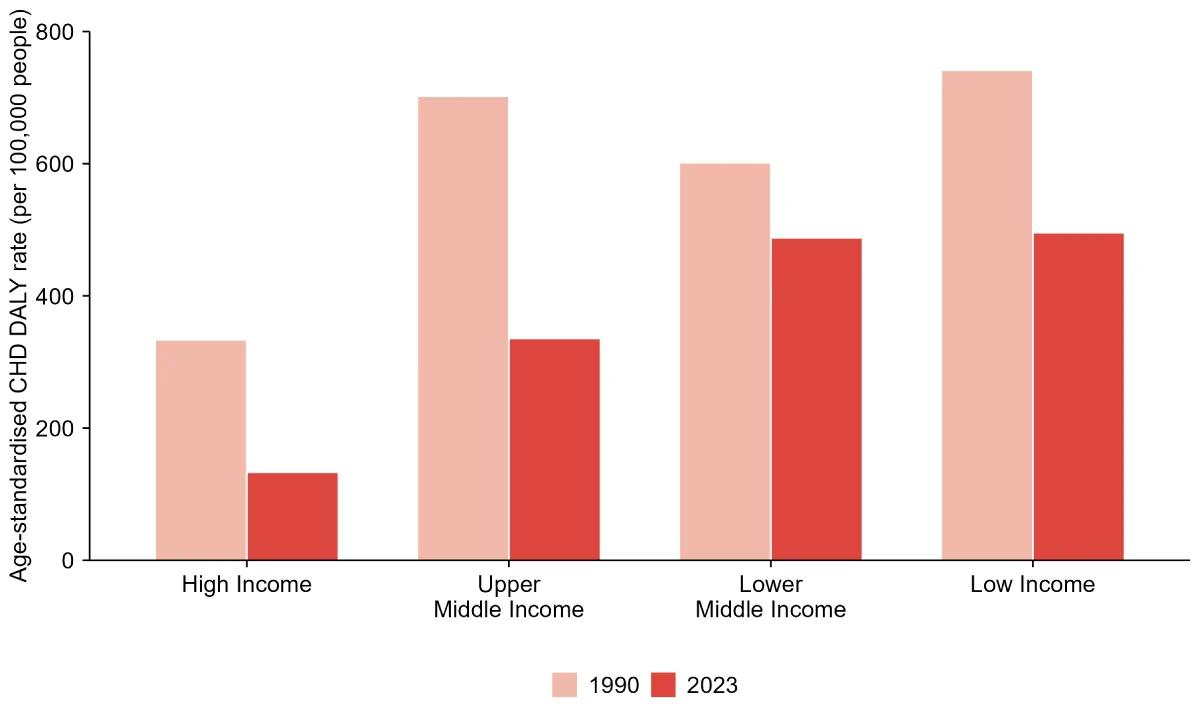
CHD age-standardised DALYs (per 100,000 people) by region, 1990 and 2023.

**Figure 8:**
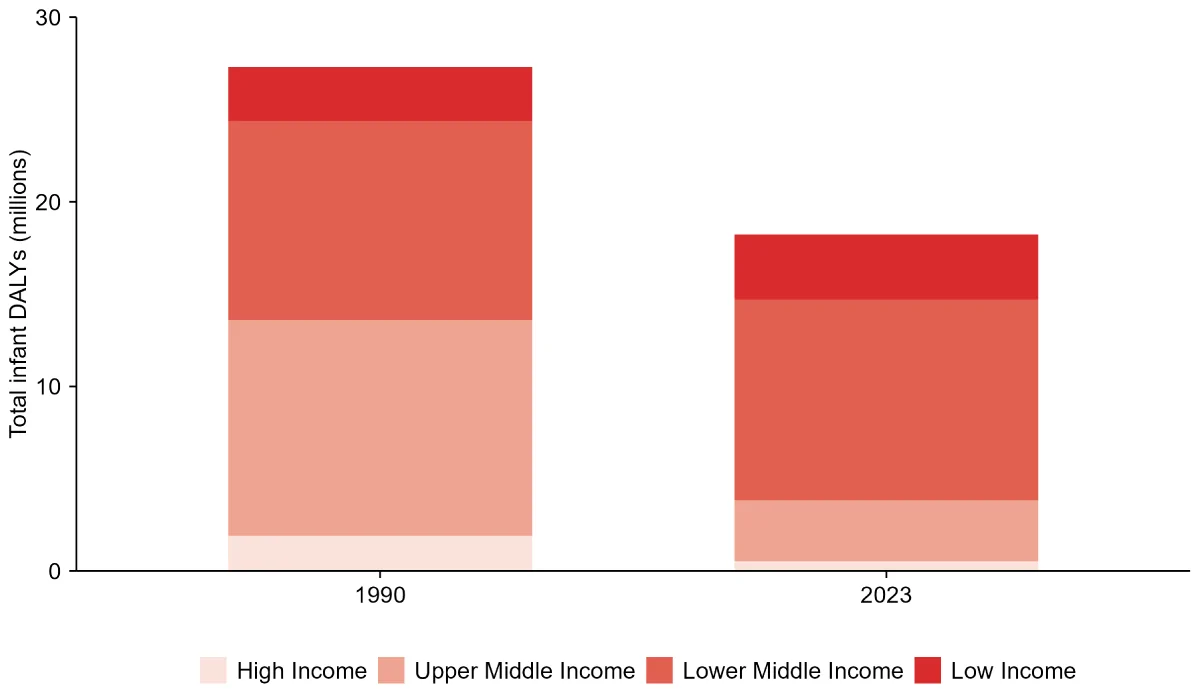
Total infant DALYs attributable to CHD by region, 1990 and 2023.

Between 1990 and 2023, the largest decline was observed in high- and upper-middle-income regions (average annual percentage change of –1.8% and –1.5%, respectively), followed by low-income regions (–1.0%) and lower-middle-income regions (–0.6%) (Figure 7). Below 1 year of age, CHD represents a high proportion of DALYs (5.8% of total DALYs). Globally, the total number of DALYs decreased by one-third, from just above 27 million to 18 million, with a reduction in contribution from upper-middle-income regions (from 42.8 to 18.3%) and a significant increase in contribution from lower-middle-income regions (from 39.5 to 59.7%) (Figure 8).

### Burden of CHD subtypes

The analysis of CHD as a whole partially limits our understanding of the burden of CHD subtypes. Evidence from the literature shows a significant degree of heterogeneity by geography and sex. Ventricular septal defect is the most prevalent subtype worldwide, accounting for 30–40% of cases, and has notable variation by ethnicity (2,12). Atrial septal defect accounts for 7–10% of all CHD, occurs more among women, and is the most commonly diagnosed CHD in adulthood (7). Patent ductus arteriosus prevalence increases at high altitudes, comprising over 60% of CHD cases in Tibetan regions above 4,000 meters (see Appendix Table 1) (13,14).

### Disease determinants: genetics versus environmental

Genetic factors play a major role, with an estimated 40% of CHD cases linked to identifiable genetic abnormalities such as chromosomal aneuploidies, copy number variants, and pathogenic mutations in single genes (15,16). Gross chromosomal anomalies account for roughly 8–10% of cases. Trisomy 21 (Down syndrome) is the most common, with about half of all newborns with this condition having CHD, most often presenting with atrioventricular septal defects.

Environmental risk factors account for about 2% of definitively attributable cases. Maternal pregestational diabetes increases the risk of CHD by 2.5- to 5-fold across multiple subtypes. Maternal obesity independently raises CHD risk by 29% (OR 1.29; 95% CI 1.22–1.37) (17). Teratogenic medications with established CHD associations include valproic acid, lithium, retinoic acid derivatives, and ACE inhibitors (17). Maternal folate status demonstrates a curvilinear dose-response relationship with CHD risk. While periconceptional folic acid supplementation reduces CHD risk by approximately 21%, recent evidence reveals that both deficiency and excess are harmful (18,19). Low maternal serum folate is associated with up to a three-fold increase in CHD risk, and excessively high levels are associated with nearly a two-fold increase. Initial evidence is emerging on the association between maternal selenium exposure and CHD with low maternal selenium status potentially associated with increased risk of CHDs in offspring (20).

Environmental and occupational exposures also contribute: organic solvents (OR 1.82; 95% CI 1.23–2.70) and maternal smoking (OR 1.16; 95% CI 1.07–1.25) are associated with increased risk at the population level (17). Regarding ambient air pollution, PM_2.5_ exposure during the second and third trimesters is associated with 23% increased CHD risk (OR 1.23; 95% CI 1.14–1.32) per 10 μg/m^3^ increase (21). Maternal exposure to extreme heat events during weeks 3–8 post-conception is associated with increased risk of CHD in offspring (OR 1.12; 95% CI 1.04–1.34), with effects most pronounced in temperate climate zones (OR 1.35; 95% CI 1.23–1.48) (22). As global temperatures continue to rise, projection models in the US estimate thousands of additional CHD cases in the coming decade. The remaining 60–70% of CHD likely arises from gene-environment interactions, where multiple genetic variants interact with environmental exposures during critical windows of cardiac development (15).

### The continuum of care

Effective CHD care depends on an integrated continuum that links community-based detection to lifelong specialist follow-up, embedded within the broader health system. When elements of this continuum are weak or fragmented, health systems struggle to deliver consistent, safe care. A well-functioning continuum requires not only clinical excellence but also multidisciplinary teams, robust learning health systems, sustained policy commitment, and adequate investment across the life course.

The continuum is best conceptualised as a tiered system, from community-based services to highly specialised children’s hospitals, each with defined capabilities. Primary health centres focus on awareness, basic screening, and timely referral; secondary facilities provide non-invasive diagnostics, stabilisation, and coordinated referral; higher-level centres offer advanced diagnostics and inpatient care; and tertiary or national children’s hospitals deliver comprehensive paediatric cardiac surgery, intensive care, and long-term follow-up. Critically, attempting high-risk interventions before establishing strong foundations at lower tiers leads to poor outcomes and erodes trust in the system (23). Such a tiered system can integrate with existing health units and systems available within each country, thus sharing resources and avoiding creating parallel efforts.

Across this continuum, care begins with recognition through antenatal screening, newborn pulse oximetry, and physical examination, followed by diagnosis and triage, referral, stabilisation and safe transport, definitive treatment, and structured follow-up. A functional system depends on a trained multidisciplinary workforce such as paediatric cardiologists, paediatric cardiac surgeons, cardiac anaesthetists, perfusionists, intensivists, and specialised nursing and allied health staff-supported by reliable echocardiography, neonatal and paediatric cardiac intensive care, safe blood supply, infection prevention, pharmacy capacity, and continuous quality monitoring (24). In many low-resource settings, the absence of even a single component can undermine an entire programme, rendering technically feasible surgery ineffective. Delays in diagnosis, late presentation, malnutrition, and sepsis further elevate operative risk, such that procedures in low-resource settings carry substantially higher mortality than in high-income countries, where survival typically exceeds 95% (5,25). CHD care does not end at surgical discharge because patients require lifelong cardiology review, management of residual disease, reproductive and mental health support, and planned transition from paediatric to adult services. Yet the capacity to deliver this continuum is unequal across the world. Inequities in the availability and quality of CHD care arise primarily from the unequal distribution of resources across health systems, spanning accessibility, capacity, quality, and affordability. Specialised paediatric cardiac centres are concentrated in high-income regions. Sub-Saharan Africa has approximately one centre per 13 million people, compared to one per 0.6 million across North America, Europe, and Central Asia. High-income countries have 140–180 times as many cardiac surgeons as low-income countries, and in Africa the median density of paediatric cardiothoracic surgeons is 0.04 per million against an international recommendation of 1.25 (26,27).

Service availability also varies systematically by income setting. Newborn and prenatal screening are standard in most high-income countries but absent or limited in low- and lower-middle-income regions as secure infant transport is inconsistent or absent outside high-income settings, and access to infant heart surgery ranges from over 80% in high-income countries to a median of 15% (range 3 to 50%) in low- and middle-income regions and 0 to 5% in low-income regions. Consequently, most children with CHD in low-resource settings remain undiagnosed and die prematurely, or present late with advanced, sometimes inoperable disease. Workforce shortages are the most critical bottleneck, compounded by infrastructure constraints such as unreliable power, limited biomedical engineering capacity, and fragile supply chains, that magnify inequities in these highly specialised services (28). It is imperative that the global movement of the workforce, especially towards the high-income nations, acknowledges and accounts for the effects of such brain drain on low- to middle-income countries.

## Discussion

This manuscript presents a comprehensive analysis of the World Heart Report 2026 on congenital heart disease, documenting a condition that represents a critical yet overlooked dimension of global cardiovascular health. Our findings reveal that the number of people living with CHD has increased by 35% since 1990, reflecting real progress in survival, however, mortality rates continue to remain high in many regions. There has been persistence of four-fold CHD mortality differences between low-income and high-income regions, showing that technical knowledge exists, but a collective lack of moral imagination (29), has failed to diffuse that knowledge globally (5). CHD continues to contribute to infant mortality despite global declines in infectious diseases, showing a burden that has historically been masked by competing causes of death. The growing levels of CHD in lower-middle-income regions, alongside increasing survival into adulthood, signals an emerging challenge for health systems that are often not designed to deliver lifelong, specialized care. Taken together, these findings suggest that persistent disparities in CHD outcomes are driven less by a lack of clinical knowledge than by unequal access to timely diagnosis, treatment, and long-term follow-up.

Effective management of CHD requires seamless coordination across multiple stages, from prenatal detection through lifelong adult care. Failure or weakness at any point undermines the entire pathway. For example, Peru’s experience with pulse oximetry screening illustrates this clearly. Without treatment capacity, detecting critical CHD shifts diagnosis from being life-saving to merely informative, placing families in the untenable position of knowing their child’s condition but being unable to access care (30). This reveals why isolated interventions such as training individual surgeons, donating equipment, or conducting short-term surgical missions produce minimal population-level impact without comprehensive system building, potentially impairing long-term benefits (31,32).

The distinction between minor, moderate, and critical CHD carries profound implications for planning. Minor lesions often require only periodic monitoring, deliverable at primary care levels. Critical CHD requires intervention in the neonatal period to prevent death, necessitating the full tertiary care ecosystem including multidisciplinary teams, reliable infrastructure, safe transport systems, and highly specialized surgical programs. Most low-income countries lack this ecosystem entirely. Many middle-income countries have it only in capital cities, creating a geographic lottery where survival depends on proximity to expertise. Furthermore, corrective repairs restore normal physiology and enable decades of life without major interventions, while palliative procedures leave patients with residual abnormalities requiring ongoing management. What matters is the ability to deliver appropriate procedures at the right time with acceptable outcomes and sustained follow-up.

Implementation of coordinated interventions at national and regional levels has demonstrated that meaningful improvements are achievable. For example, Kerala’s Hridyam program in India has shown how integrating CHD screening into maternal and child health services, establishing referral systems, and providing public financing can improve detection and access to care not only for CHD but also for non-CHD-related infant mortality (32,33). Similarly, initiatives such as the International Quality Improvement Collaborative illustrate that systematic data collection, benchmarking, and peer learning can reduce mortality even in resource-constrained settings, with outcomes approaching those observed in high-income countries for several conditions (33,34,35,36,37).

The economic burden of CHD varies substantially across contexts but remains considerable everywhere. In the United States, lifetime costs for complex CHD average $2.1 million per patient, with families bearing an average of $190,000 out-of-pocket (38). In low- and middle-income countries, while absolute costs are lower, they often represent catastrophic expenditure relative to household income. In Rwanda, surgical costs averaging $7,700 exceed eight times the country’s per capita GDP (39). In India, families frequently resort to asset sales or loans to fund care (40). Beyond direct medical costs, indirect consequences including caregiver productivity losses and foregone economic contributions from premature death substantially increase societal burden.

Workforce development represents the most critical bottleneck limiting global CHD care. Sub-Saharan Africa has one paediatric cardiac surgery centre per 13 million people, while North America and Europe have one per 0.6 million (26). Training a paediatric cardiac surgeon requires 10–15 years beyond medical school, and most training occurs in high-income countries. Retention in low- and middle-income countries fails due to inadequate infrastructure, overwhelming demand, and salary differentials (28,41). Task-sharing strategies, where nurses and non-physician providers are trained for specific roles like focused echocardiography or stable patient follow-up, can extend reach when implemented with appropriate training and supervision (42). Technology offers additional opportunities, with telemedicine linking rural providers to urban specialists and digital tools supporting patient tracking, though implementation must avoid exacerbating digital divides. However, the introduction of digital/low-cost solutions requires real-world evidence of effectiveness on health outcomes (43).

Beyond survival, CHD imposes a substantial and under-recognized burden across the life course. Women with CHD face unique challenges related to reproductive health, including risks during pregnancy and the need for specialized cardio-obstetric care (8,9). However, access to appropriate counselling and multidisciplinary services remains limited, particularly in low- and middle-income countries, contributing to preventable maternal morbidity and mortality (44). Similarly, the mental health burden associated with CHD is considerable but frequently overlooked. A significant proportion of children experience neurodevelopmental or psychological conditions, with effects extending into adulthood, including educational challenges, reduced employment opportunities, and diminished quality of life (45,46). Families are also affected, often experiencing psychological distress and financial strain (47,48). Despite this, mental health services are rarely integrated into CHD care pathways, and available evidence is largely derived from high-income settings.

The multifactorial aetiology of CHD presents both challenges and opportunities for prevention. While a substantial proportion of cases is linked to genetic factors, modifiable maternal and environmental exposures play an important role (17). Evidence linking maternal diabetes, obesity, smoking, medication exposure, and folate imbalance to CHD risk supports the potential for targeted prevention strategies (18,19). Emerging data on ambient air pollution and extreme heat exposure further expand the scope of preventable risk factors, suggesting that environmental and climate-related policies may contribute to reducing CHD incidence (21,22). However, these pathways remain complex, and further research is needed to better characterize gene-environment interactions and identify effective interventions.

CHD’s marginality within global health agendas reflects multiple factors including lack of epidemic potential and powerful advocacy constituencies. Breaking this cycle requires strategic reframing. CHD must be positioned within child survival frameworks, with targets integrated into universal health coverage and Sustainable Development Goal monitoring (49). The proposed 2027 World Health Assembly resolution on childhood-onset heart disease care would create accountability mechanisms that are currently absent (50).

This analysis has several limitations. Estimates rely heavily on GBD data, which may be subject to uncertainty in regions with limited surveillance systems. The true burden of CHD is likely underestimated in under-resourced settings. In addition, a focus on mortality and DALYs may not fully capture the broader psychosocial and functional impacts of CHD. Evidence gaps remain, particularly in relation to mental health, gender-specific outcomes, and optimal models of care in low-resource environments.

In conclusion, CHD burden is substantial, growing, and profoundly inequitable. Effective interventions exist, but political will and a lack of moral imagination, rather than technical barriers explain persistent gaps. National CHD strategies integrated within broader health planning must define targets, allocate resources, and establish accountability. These strategies must address the full continuum from prevention through adult management, requiring contributions across multiple sectors. Professional societies like the World Heart Federation offer platforms to scale efforts and amplify patient voices. The biomedical industry must engage through tiered pricing and technology transfer to ensure access regardless of ability to pay with governance and accountability driven through WHO/WHA. Addressing the global CHD crisis requires coordinated efforts from policymakers, healthcare systems, and international organizations. The World Heart Federation calls for urgent collective action to reduce CHD mortality and morbidity through comprehensive, evidence-informed strategies. The World Heart Federation recommendations include:

1. All countries should urgently increase national-level capacity across the health system to care for people with childhood-onset heart disease, including CHD. This includes developing and scaling centres of excellence, improving referral networks from early detection and diagnosis to surgery, long-term follow-up, and transition to adult care, and integrating congenital heart services into broader maternal, newborn, and child health systems. Ultimately, the goal is to move from episodic, acute care to sustainable, locally led systems capable of delivering timely, high-quality population-level services ensuring continuity of care across the lifespan. Such programmes require sustained investment with inclusion of CHD services into UHC benefits packages to avoid financial hardship or catastrophic costs for affected families.
2. Countries should invest in training and building the paediatric and congenital cardiac workforce and strengthening capacity of the adult CHD workforce. This includes training and retaining multidisciplinary specialized paediatric and congenital cardiac care teams and requires developing national CHD health workforce development plans based on population needs forecasting that include development of formal training pathways.
3. Policymakers at international and national level must ensure CHD is reflected in relevant policy frameworks. NCD, maternal and child health and surgical policy initiatives should include measures to improve access to, and quality of, CHD care across the care continuum. This can further support the aims of such policies to reduce NCD and infant mortality, in alignment with the Sustainable Development Goals. Furthermore, policymakers should support the development of national, context-appropriate guidelines to improve implementation of best practice and CHD outcomes.
4. Countries and international bodies should work together to improve CHD surveillance and close the data gap to enhance the understanding of CHD epidemiology and determinants. This requires strengthening national health information systems to capture data on CHD prevalence, outcomes, and service delivery, such as through population level registries. Such data should include more granular and comparable information on subtypes of CHD. Better data would enable countries to improve care delivery based on quality improvement metrics, plan and allocate resources more effectively, and track progress over time.
5. Civil society, including national NCD and CVD organizations, should support the campaign for a 2027 WHA Resolution on Childhood-Onset Heart Disease. The resolution will help achieve Universal Health Coverage and reductions in preventable deaths of newborns and children under five, by ensuring countries have paediatric and congenital cardiac care as an integral part of the national health system. Advocates can use resources provided by the Global Coalition for Pediatric and Congenital Hearts to plan and implement national campaigns.
6. Advocates for CHD care should work to amplify the voices of CHD patients and their families to drive efforts forward. Lived experiences provide unique insights that can inform service delivery, research priorities, and support systems. Empowering patient organisations fosters a patient-centred approach to care and ensures that the needs of those affected are at the forefront of global efforts.

## Appendix

**Appendix Table 1:** Global distribution of common congenital heart disease subtypes.

CHD SUBTYPE	GLOBAL PREVALENCE	COUNTRIES/REGIONS WITH NOTABLE PATTERNS	EXAMPLES (KEY STUDIES)

**Ventricular septal defect (VSD)**	30–40% of all CHD	Commonest VSD worldwide. Subpulmonic VSD common in China, Japan, Taiwan, Thailand, Korea.	China: VSD 3.3/1,000 live births; Northwestern China 29.2%; Jinan 1.18/1,000 live births. Nigeria: 40.6% of all CHD. Uganda: 27.2%. India: North 5.7/1,000; Mumbai 42.86%. Australia: 25%.

**Atrial septal defect (ASD)**	7–10% of all CHD	Commonest CHD diagnosed in adulthood. Secundum ASD accounts for 80% of ASDs. More common in women.	Australia: 10%. China: Jinan 3.07/1,000 live births. Uganda: 9.4%, 88% secundum.

**Patent ductus arteriosus (PDA)**	<10% of CHD	Higher at high altitudes.	Tibet, Nagqu (4,200–4,900 m): 66.3% of all CHD. Ngamring: 55.42%. Tanzania: 19.1%.

**Tetralogy of Fallot (TOF)**	0.3–0.5 per 1,000 live births; 3–5% of all CHD	Most common cyanotic CHD.	UK: 6.5%. India (Uttarakhand): 5.45%. Nigeria: 7.8%. Africa: 0.52/1,000 live births.

**Transposition of the great arteries (TGA)**	0.2–0.4 per 1,000 live births; 4–5% of all CHD	Male predominance is universal. Limited survival data in LMICs owing to lack of neonatal surgery.	—

CHD, congenital heart disease; LMICs, low- and middle-income countries.
